# Measuring the quality and quantity of professional intrapartum support: testing a computerised systematic observation tool in the clinical setting

**DOI:** 10.1186/1471-2393-13-163

**Published:** 2013-08-14

**Authors:** Mary C Ross-Davie, Helen Cheyne, Catherine Niven

**Affiliations:** 1 Educational Projects Manager, Midwifery and Reproductive Health, NHS Education for Scotland, Westport 102Westport, Edinburgh EH3 9DN, UK; 2 Royal College of Midwives Professor of Midwifery & Professor of Maternal and Child Health Research, NMAHP Research Unit, University of Stirling, Stirling FK9 4LA, UK; 3 Emeritus Professor, University of Stirling, Stirling FK9 4LA, UK

**Keywords:** Intrapartum, Labour, Support, Midwife, Systematic, Observation, Quantity, Quality, Clinical outcomes

## Abstract

**Background:**

Continuous support in labour has a significant impact on a range of clinical outcomes, though whether the quality and quantity of support behaviours affects the strength of this impact has not yet been established. To identify the quality and quantity of support, a reliable means of measurement is needed. To this end, a new computerised systematic observation tool, the ‘SMILI’ (Supportive Midwifery in Labour Instrument) was developed.

The aim of the study was to test the validity and usability of the ‘Supportive Midwifery in Labour Instrument’ (SMILI) and to test the feasibility and acceptability of the systematic observation approach in the clinical intrapartum setting.

**Methods:**

Systematic observation was combined with a postnatal questionnaire and the collection of data about clinical processes and outcomes for each observed labour.

The setting for the study was four National Health Service maternity units in Scotland, UK. Participants in this study were forty five midwives and forty four women.

The SMILI was used by trained midwife observers to record labour care provided by midwives. Observations were undertaken for an average of two hours and seventeen minutes during the active first stage of labour and, in 18 cases, the observation included the second stage of labour. Content validity of the instrument was tested by the observers, noting the extent to which the SMILI facilitated the recording of all key aspects of labour care and interactions. Construct validity was tested through exploration of correlations between the data recorded and women’s feelings about the support they received. Feasibility and usability data were recorded following each observation by the observer. Internal reliability and construct validity were tested through statistical analysis of the data.

**Results:**

One hundred and four hours of labour care were observed and recorded using the SMILI during forty nine labour episodes.

**Conclusion:**

The SMILI was found to be a valid and reliable instrument in the intrapartum setting in which it was tested. The study identified that the SMILI could be used to test correlations between the quantity and quality of support and outcomes. The systematic observational approach was found to be an acceptable and feasible method of enquiry.

## Background

Intrapartum support has been defined as:

‘… *non*-*medical care that is intended to ease a woman*’*s anxiety*, *discomfort*, *loneliness and exhaustion*, *to help her draw on her own strengths and to ensure that her needs and wishes are known and respected*. *It includes physical comfort measures*, *emotional support*, *information and instruction*, *advocacy and support for the partner*’ p721 [1].

Theories of social support and its role in improving health are well developed [2-7] and a significant number of randomised controlled trials have identified a link between continuous support for women in labour and a range of improved outcomes [8].

A consensus exists in the literature defining three sub-categories of labour support: emotional, physical and informational support [9-12]. Emotional support is defined as expressions of love, admiration, liking, reassurance and respect, spending time with the client and making them feel cared for. Physical support includes direct assistance and informational support includes advice, information and feedback [6,7]. Over time, the categories of advocacy and partner support have been added in some studies to reflect research findings [8].

Continuous support during labour and childbirth is a key factor in shaping birth outcomes including type of birth, analgesia used, length of labour, neonatal Apgar scores and women’s feelings about their birth experience. A meta-analysis of randomised controlled trials comparing continuous with intermittent support has identified a variable effect on outcomes [8]. It has not been clear from existing studies whether the strength of the effect of the support on outcomes is linked to the quantity and quality of support behaviours. Research is required to study the relationships between the content of support and outcomes. However, a method of enquiry is first required that will enable the quality and quantity of supportive behaviours to be measured reliably. One such method is systematic observation.

Systematic observational research is a research methodology first developed in behavioural psychology [13-16]. The data collection instrument in such research is the observation instrument, a paper or computer based checklist, that enables an observer to record systematically the behaviours of interest. A review of earlier systematic observation instruments for the measurement of intrapartum professional support [11,17-20] identified significant gaps, with some key elements of support, including the quality of the support, not measured. Therefore it appeared that a new systematic observation instrument was required for research in intrapartum support. In developing and testing an instrument for systematic observation of intrapartum care a number of methodological and ethical challenges need to be considered: labour onset and progress are unpredictable, complicating forward planning and time management for the research; direct observation is a time consuming and labour intensive method of enquiry; labour is an emotional and stressful time for the woman, her birth partner and her professional carers and this raises concerns about the acceptability of introducing an observer into the labour room and the timing of consent procedures.

A computer based systematic observation instrument, the ‘Supportive Midwifery in Labour Instrument’ or ‘SMILI’, was developed, based on evidence about the key elements of high quality intrapartum support [9,21-33]. The SMILI enables a trained observer, present in the labour room, to record the woman, birth partner’s and midwife’s demeanour, words and actions intermittently during active labour and to record the movement of the midwife and others in and out of the labour room. The development of the ‘SMILI’ is described in detail elsewhere [34]. More than 90 possible options to describe the midwife’s demeanour and behaviours were available for the observer to record every three minutes. The SMILI was successfully tested for face and content validity and inter- and intra-rater reliability using video recordings of labour scenarios [34]. However, before use, the instrument required to be further tested in the real life setting. This paper describes the pilot and feasibility testing of the SMILI in a clinical setting.

### Aim

The aim of the study was to test the validity and reliability of the SMILI and its feasibility in the intrapartum setting.

To ensure that data produced by any new instrument have meaning and can be accurately interpreted and applied, it is necessary to thoroughly test for validity and reliability. Face, content and construct validity cannot be demonstrated through one test, but rather are shown through a series of tests that highlight the relationship between the instrument and the behaviour it is intended to measure [35,36]. An instrument is said to have face validity when it appears to a group of experts to be able to measure the behaviours of interest and has content validity when the items included in the instrument are considered to represent the entire range of possible items the instrument should cover [35-38]. Construct validity describes the association between the data collected and the prediction of a theoretical trait or construct [39-41]. Reliability relates to the consistency of a measure. A measure is considered reliable if the same result would be produced when different raters use the measure or if the measure was repeated on different occasions [35,42]. Internal reliability measures the consistency of the data measured by the instrument, identifying if related items correlate with each other [43]. Feasibility refers to the extent to which the instrument or study design can be successfully employed in a real life setting [36].

### Objectives

The study objectives were:

1. To test the feasibility and acceptability of the systematic observational approach and the SMILI in the intrapartum clinical setting.

2. To test the SMILI for content and construct validity and internal reliability.

3. To explore the ability of the SMILI and birth outcome measures to measure the quality and quantity of midwifery support in labour in the clinical setting.

## Methods

### Study design

Quantitative methods were used. The research design combined systematic observation of clinical care, a postnatal questionnaire for women participants and collection of clinical processes and outcome data for each observed labour.

Feasibility was assessed by self-report from the observers, the midwives and women observed through completion of a postnatal questionnaire. This assessed ease of recruitment of participants, the acceptability of the observation process to midwives and women. Observers also commented on the usability of the instrument in the real life setting and noted any gaps in the data they were able to record.

Testing of construct validity was undertaken through the identification of correlation coefficients between the behaviour variables recorded and women’s and observers’ views of the support received or observed. If the SMILI was not effectively measuring the central construct of support, the data would not correlate significantly with women’s and observers’ overall assessments of the midwifery support. The internal reliability or consistency of the SMILI was tested through the exploration of the relationship between groups of variables that would be expected to be related.

### Setting

The study was conducted in four diverse maternity units in NHS Scotland, these were: a midwife-led unit offering intrapartum care to low risk women and three consultant-led units one of which had an alongside midwife-led unit and one without the facility of an intrapartum epidural service.

### Ethical considerations

The study received ethical approval from the University of Stirling Department of Nursing and Midwifery research ethics committee and Tayside B NHS research ethics committee for NHS Scotland. Research and development approval from all four participating units was received.

### Participants

To obtain the required data, three groups of participant were required: the observers, the midwives being observed and the women and their partners being cared for in labour.

The four observers were the principal investigator and a sample of three experienced clinical midwives working for NHS Scotland who responded to a request for volunteers circulated via a professional network.

The sample of midwives was drawn from the midwives who were on duty to provide intrapartum care at the time of the researcher’s attendance in the unit. Midwives were given an explanation of the study and were asked to give written consent. Forty five midwives participated in the study. Four midwives allowed observers to be present on two occasions when they were caring for different women. All of the midwives observed were caring for only one woman throughout the observation period without responsibilities towards any other women on the unit. Key characteristics of the midwives observed for the study are given in Table 1.

**Table 1:** The midwife participants

**Midwife characteristics n** = **45**	**Categories**	**Number**	**Percentage**
Ethnic Origin	White European	44	97.7
	Asian	1	2.3
Training/Education	Pre-registration long course (‘direct entry midwife’)	26	57.8
	Short course (dual qualified nurse and midwife)	19	42.2
Experience in years	0-5	11	24.4
	6-10	8	17.8
	11-15	6	13.3
	16-20	7	15.6
	>20	13	28.9
Age in years	20-25	1	2.2
	26-30	6	13.3
	31-40	13	28.9
	41-50	11	24.4
	51-65	14	31.2
Working hours	Part time	24	53.3
	Full-time	21	46.7
Number of women allocated to care for	One woman	45	100
	>1	0	0

The study was based on observation of the care of women during labour. This creates some ethical challenges in providing participants with a ‘cooling off period’ between information giving and consent and seeking consent at a time characterised by emotional stress. An approach was devised that sought to minimise these ethical challenges as far as possible while also ensuring a feasible recruitment strategy. Women were given an information leaflet about the study by their midwives from 36 weeks of pregnancy and posters about the study were displayed prominently in antenatal clinic areas in participating units in the run up to the recruitment period and during the data collection period. Further information and gathering of written consent was undertaken by midwives working on the maternity unit upon admission for induction of labour or in early spontaneous labour. Women and their birth partner were given an explanation of the study and an information leaflet, which they were then left to consider for around one hour before consent to participate was sought. The researchers were not involved in any part of the consent process to reduce any feelings of pressure to consent. Women were eligible to participate in the study if they were experiencing a healthy term pregnancy and were in early active labour at a time when the researcher was in attendance at the maternity unit. Women were excluded if they were aged under 16 years, had a learning difficulty or mental health problem that precluded her from providing informed consent and any woman who was known to be carrying a baby likely to experience significant medical problems. The midwives were asked not to discuss participation if a woman was distressed. As part of the consent procedure, women and their birth partners were advised that they could change their mind about participation at any point and simply ask to speak to the midwife privately to inform them that they would like the observer to leave. Forty four women and their birth partners participated in the study, five women agreed to have an observer present for two sequential observations, when they were cared for by two different midwives during their labours. Key characteristics of the woman and birth partner participants are given in Table 2.

**Table 2:** The women and birth partner participants

**Characteristics of woman and birth partner participants**	**Categories**	**Number**	**Percentage**
Number of women observed		44	
Women observed on two occasions		5	
English woman’s first language		44	100
Woman’s ethnic origin	White European	44	100
Cervical dilatation at last Vaginal Examination before observation commenced (n = 49)	</=3cm	12	24.5
	3-5cm	20	40.8
	>5cm	16	32.7
	Unknown	1	2.0
Allocated care pathway (n = 49)	Red (high risk)	19	38.8
	Amber	8	16.3
	Green (low risk, midwife-led)	22	44.9
Woman Age (n = 44)	<20	7	15.9
	20-25	13	29.5
	26-35	16	36.4
	>35	8	18.2
Woman’s Parity (n = 44)	0	25	56.8
	1	9	20.4
	2	5	11.4
	3+	5	11.4
Birth partner present (n = 44)	44 (100%)	44	100
	Life partner only birth partner	34	77.3
	Mother only partner	6	13.6
	Life partner + mother	4	9.1

### Sample size

Decisions about the sample size for this study were based on a review of studies employing a similar methodology [44]. A sample of fifty labour care observation episodes was selected. If observations were two to three hours long this would represent 100–150 hours of observation. This would ensure that the sample was of a similar size to the most comparable systematic observation studies in maternity and non-maternity settings [11,17-20].

### The instruments and data collection procedures

The observers used the ‘SMILI’ to record the labour care observed. More than 90 possible options to describe the midwife’s demeanour and behaviours were available for the observer to record every three minutes.

Women’s views of the support they received during labour were captured through completion of the validated ‘Support and Control in Birth (SCIB)’ questionnaire [45]. The SCIB is a 33 item questionnaire with a 5-point response scale (1 to 5) with high scores indicating more support or control. The questionnaire has three subscales:

1. *Internal Control*, 10 items, average score calculated, range 1 – 5.

2. *External Control*, 11 items, average score calculated, range 1–5.

3. *Support*, 12 items, average score calculated, range 1 – 5.

Total scores range from 3 to 15. The average scores for each woman who completed the SCIB were calculated for each of the three subscales. A score of 5 indicates the maximum possible score and 1 the lowest [45]. The SCIB score for the subscale of support is the score considered in this paper.

The observers’ global assessments of the quantity and quality of the support were assessed using two Likert scale responses included in the postnatal data outcomes sheet. For each observation global observer scores were given for the quantity and quality of the support observed from 0 (poor support observed) to 4 (excellent). These responses were included in the next stages of the analysis to identify whether the assessment by the observer corresponded with the results recorded in the SMILI and with women’s assessment of care recorded in the SCIB questionnaire. The comparison of the observer and women’s assessments of the support functions as a further test of the instrument’s validity and ‘veridicality’. Veridicality refers to the level of congruence between the perceptions of support of the recipients and someone else, in this case, the observers, who may be considered experts in intrapartum midwifery support [46].

The Postnatal outcomes data sheet allowed the recording of several key labour processes and outcomes for each labour where observation was undertaken. This included the type of pain relief used during the labour as a whole, the type of medical interventions employed and the type of birth. The postnatal outcomes data sheet, designed specifically for this study, was completed by the observer.

### Data analysis

The 90 observable positive and negative behaviours were grouped into eleven categories to facilitate analysis. The eleven categories were emotional support, informational support, tangible support, advocacy, support of the partner, assessment, non support direct care, indirect care, lack of attentiveness, neutral/professional attitude and negative/authoritarian behaviour, Categories with a large number of behaviours, such as emotional support, were also grouped into sub-categories for more detailed analysis.

The quantity of support was measured in two key ways by the SMILI: recording the time that the midwife was in the room with the woman and the proportion of that time that the midwife was engaged in supportive activities. The SMILI enabled the observer to note by clicking a button when the midwife left the room and when the midwife returned. A timer in the programme recorded the number of seconds that a midwife was out of the room. This enabled the calculation of the proportion of the observation as a whole that a midwife was out of the room.

Frequency of observed behaviours were a key element in testing the content and construct validity of the SMILI. Frequency data for each behaviour were saved directly into Microsoft Excel spreadsheets. The frequency of contractions and observation points were the denominator to calculate the percentage that each behaviour was observed. It was possible for categories of behaviour to be described as being present for more than 100% of the observation as a number of different behaviours in one category could be seen within one three minute observation point (for example, within the category of verbal support, the behaviours ‘giving reassurance, praise and encouragement’, ‘showing empathy’ and ‘talking a woman through a contraction’). The data were transferred from Excel to SPSS 17 for analysis.

The assessment of whether the SMILI was successful in measuring the quality of midwifery support was undertaken in a number of ways: the comparison of the quantity of positive and negative support behaviours between midwives, the time each midwife spent in the room and the exploration of relationships between the recorded behaviours and women’s assessment of the care received. This draws on the Instititue of Medicine definition of quality in healthcare that the care should be ‘person-centred, safe, effective, efficient, equitable and timely p3 [47] and the social support theoretical framework which suggests that support may only be evaluated as being of high quality if it is considered to be so by the recipient [48].

To test the internal reliability or consistency of the SMILI it was necessary to test the relationship between related variables. This analysis seeks to identify whether there is a significant level of consistency between variables that would be expected to be related. A Cronbach’s alpha analysis was employed, using George and Mallery’s accepted definition for strength with > .8 good, >.7 acceptable and < .6 questionable [43].

The outcome data were collected to test the construct validity of the SMILI, through identifying the relationship between the outcomes and the support recorded.

The Spearman’s Rho is the appropriate statistical test for the data produced in this study as it is a non-parametric test that allows for the calculation of correlations between ordinal data (such as Likert scale results in the SCIB and postnatal data sheet responses), that is not necessarily normally distributed. The strength of the relationship between the variables is expressed as a figure between 0 and 1. The closer the figure is to 1, the stronger the correlation.

## Results

Fifty two observations of intrapartum care were observed during the four month data collection period. One hundred and eleven hours of direct intrapartum observation were undertaken. The average length of each observation was 127.7 minutes (range 45.8 minutes – 318 minutes). Data were lost in three observations due to user error. Full data were recorded and analysed for forty nine observations, 104.3 hours of observation. Observations were often shorter than the planned three hour period as women progressed quickly through labour and to the birth of their baby in one third of the observations. A summary is provided in Table 3.

**Table 3:** Overall study figures for observations

**Number of observations with complete data**	**N** **=** **49 (missing data n** **=** **3)**
Total hours of observation	104.3 hours
Average length of observation	127.7 minutes
Range of observation length	45.8- 318.0 minutes
**Number of observations by unit type and size**	
Annual births >3000 Consultant led unit	38 (77.6%)
Annual births 1500–3000 Alongside midwife led unit	8 (16.3%)
Annual births <500 Community midwifery unit	3 (6.1%)

### Feasibility and acceptability data

Verbal feedback was sought from all of the observers about their experience of being involved in the study and any problems they encountered in carrying out the direct observations in the clinical setting. All three of the volunteer observers reported feeling very positive about their involvement in the study, being surprised with the ease with which they were able to gain consent from participants and how accepted and welcomed they felt by staff.

There were no problems experienced during the data collection period in gaining access to the ward areas or to midwifery staff to explain the study and to ask them to discuss participation with women. The midwives were generally enthusiastic and positive about the study and interested in the outcomes. Following the end of the data collection period, the local collaborators at each of the sites were asked to talk informally to staff about the study and identify any problems or concerns that staff had not felt able to share with the research team. All of the feedback given to the local collaborators was positive and no concerns or problems were raised.

Data were not routinely collected of numbers and reasons for women, birth partners and midwives not wishing to participate. Verbal and written information about the study was given to all midwives providing labour care to eligible women on a shift when an observer was present. Information was provided and consent sought from the woman and birth partner either by the midwife caring for her in labour, the midwife caring for her in the triage unit or the coordinator of the labour ward. Once consent had been provided by a woman and her birth partner to participate in the study, the researcher undertook the consent procedure with the midwife caring for her.

The midwife and woman participants responded positively to a postnatal question about the presence of an observer during the labour. Twenty nine midwives (64.4%) agreed with a statement that they felt ‘fine, enjoyed it’, with sixteen (35.6%) stating that the experience of being observed was ‘OK’. No midwives chose the negative options of ‘distressed, very or mildly uncomfortable’. Forty four of the midwives stated that they would participate again. Thirty nine women (88.6%) felt that having an observer was ‘fine’ and 11.4% chose ‘OK’. All of the women involved in the study said they would be happy to participate again in a similar study.

### Face and content validity and usability of the SMILI in the clinical setting

The responses in the postnatal data collection sheet demonstrated that the SMILI had content validity with only a few problems and gaps identified in the earlier observations. In 28 observations (57.2%) the observer felt that the SMILI enabled them to record the midwifery support they observed ‘fully’ and in 19 observations (38.8%) ‘very well’. In two observations the observers stated that the SMILI was inadequate, these were occasions when the programme temporarily crashed. Additions were made as a result of the comments and no further problems were identified after these changes were made. Problems with the programme were rare during the 111 hours of observation and overall the programme proved very reliable and usable in the clinical setting.

### Internal reliability of the SMILI

Internal reliability was found to be acceptable to good [43], with the exception of the negative variables of the woman and partner which show a weaker correlation. The results are summarised below in Table 4.

**Table 4:** Internal consistency of the SMILI

**Variables tested for internal consistency with one another**	**Cronbach’s alpha**
Woman – negative demeanour, neutral demeanour, negative and neutral vocal, negative and neutral facial	.563
Woman – positive demeanour, positive vocal, positive facial	.857
Partner – negative demeanour, neutral demeanour, far from woman, negative verbal, neutral verbal, negative facial, neutral facial, not touching, ignoring woman	.449
Partner – positive demeanour, next to woman, positive verbal, positive facial, supportive touch, engaging with woman	.836
Midwife – negative and neutral demeanour, far from woman, negative and neutral vocal, negative and neutral facial, proportion out of room	.688
Midwife – positive demeanour, next to or near woman, positive vocal and facial	.840
Midwife - positive demeanour, next to or near woman, positive vocal and facial and emotional support	.710

### Content and construct validity testing: using the SMILI to measure the quantity of support

Most midwives (92%) were in the room for more than 80% of the observation, with around one quarter of midwives present for 98% of the observation.

The overall results for the forty nine observations were:

•The total observation time with complete data was 104.3 hours.

•The mean length of each observation was 127.7 minutes. Observations ranged from 45.8 minutes to 318 minutes in length.

•The mean length of time that a midwife was out of the room excluding breaks was 11.5 minutes, which is 9.3% of the observation time. This ranged from 0% to 33.8% of the observation.

•The average number of times that the midwife left the room was six.

•Midwives left the room on average every 25.7 minutes, with a range from every 9.6 minutes to 165 minutes without leaving the room.

### The quantity of positive support behaviours

The quantity of supportive behaviours varied considerably between the midwives observed. The most frequently observed category of support was emotional support, with a study mean of 395.5% (that is an average of approximately four emotionally supportive behaviours displayed at each observation point across the study as a whole). The lowest frequency that emotional support was observed was 98.9% in one observation (an average of just below one emotionally supportive behaviour at each observation point) and the highest frequency demonstrated was 629.7% (that is more than six emotionally supportive behaviours observed at each observation point). The second most frequently observed category was informational support, followed by tangible support and then partner support. These results are summarised in Table 5.

**Table 5:** **Quantities of categories of support behaviours for overall study**, **n** = **49**

**Behaviour category**	**Study mean %**	**Standard deviation**	**Lowest frequency %**	**Highest frequency %**
Emotional Support	395.5	109.2	98.9	629.7
Informational Support	38.9	19.1	6	93
Advocacy	0.2	0.7	0	3.6
Tangible support	18.8	11.2	3.3	56.8
Partner support	7.5	7.3	0	35.7

Both emotional and informational support were relatively normally distributed. Advocacy, tangible and partner support were not distributed normally and were skewed in frequency to the less frequent.

### The quantities of neutral/professional and negative behaviour categories

The quantities of neutral/professional and negative behaviours also varied significantly between the midwives in the study. The frequency data for individual midwives revealed some patterns of behaviour. There were nine midwives who had below and two above study average frequency of all of the neutral and negative behaviours. In a similar manner to positive support behaviours, the majority of midwives (n = 37, 75.5%) showed a mixture of behaviours, displaying some neutral or negative behaviours. The quantities of neutral/professional and negative behaviours by the midwives are further summarised in Table 6. Overall, negative behaviours were seen infrequently.

**Table 6:** **Quantity of neutral**/**professional and negative behaviours**

**Behaviour**	**Study mean**	**Standard deviation**	**Lowest frequency**	**Highest frequency**
Neutral professional demeanour	37.7	29.3	1.6	122.2
Lack of attentiveness	26.2	26.4	0	112.7
Lack of proximity	6.9	6.9	0	31.6
Negative behaviours	11.6	16.3	0	101.4
Negative demeanour	2.6	12.1	0	83.9
Negative emotional	3.2	2.0	0	10.7
Negative tangible	0.3	1.5	0	10.7
Negative partner	0.09	0.3	0	1.9
Negative Information	1.3	2.8	0	15
Negative taking control	3.9	6.3	0	34.1

These results not only relate to the quantity of supportive and non-supportive behaviours observed and recorded but also contribute to the analysis of the quality of the support observed.

### Content and construct validity testing: the measurement of the quality of intrapartum midwifery support

This was calculated using the Spearman’s Rho correlation coefficient test.

The postnatal ‘SCIB’ questionnaire was completed fully for 42 of the 49 observations (86%) Where a woman was observed being cared for by two different midwives she was asked to complete two SCIB questionnaires. Women generally reported feeling very well supported in the questionnaire. The mean score for the study was 4.6 out of a possible total of 5.

The observers’ global assessments of the quality and quantity of care observed were also generally skewed to the positive (Table 7).

**Table 7:** Observer global assessments of support

**Global observer assessment question**	**Response**	**Frequency(n=** **49)**
The quantity of midwifery support I observed was	Poor	0
	Adequate	3 (6.2%)
	Good	7 (14.2%)
	Very good	20 (40.8%)
	Excellent	19 (38.8%)
The quality of midwifery support I observed was	Poor	3 (6.1%)
	Adequate	2 (4.1%)
	Good	9 (18.3%)
	Very good	19 (38.8%)
	Excellent	16 (32.7%)

Spearman’s Rho correlation coefficients were calculated between rates of negative behaviours by the midwife recorded in the SMILI and the assessments of support recorded by the woman in the SCIB and the observer in the global ratings questionnaire. Negative behaviours and inattentiveness by the midwife showed significant moderate inverse correlation with the assessment of the midwifery support by women and observers (Table 8).

**Table 8:** Correlations between negative behaviours and inattentiveness and assessment of support

**Midwife behaviours**	**Spearman’s rho**	**Woman’s assessment SCIB support**	**Observer’s global rating of quality**
Negative behaviours	Correlation coefficient	-.311*	-.385**
	P value	.024	.006
Inattentiveness	Correlation coefficient	-.284*	-.500**
	p value	.036	.000
Proportion out of room	Correlation coefficient	-.503**	-.516**
	Sig (1 tailed) P value	.001	.002

(** = Significance at p value of <0.01, * = Significance at p value of <0.05).

One of the key elements of support derived from the literature is the importance of the continual physical presence of the midwife to woman’s feelings of being supported. The results of the analysis to test the association between these elements are given below. This analysis sought to test whether the quantity of presence or attendance by the midwife is an element in the assessment of the quality of the support. These results (presented in Table 8 below) show a significant strong inverse correlation between the amount of time that the midwife spent out of the room and the woman and observer’s assessments of support. The higher the proportion of the observation that the midwife was out of the room, the lower the assessment of the support offered.

Correlation coefficients were calculated between positive midwifery support behaviours and the assessments of support by the woman and observer, in order to test whether the quantity of positive midwifery support behaviours is a key element in the measurement of the quality of midwifery support. These results show a significant moderate correlation between women’s assessment of the support they received and the overall measurement of emotional support and support of the partner (Table 9). Analysis of the data for the sub-categories of emotional support found that the most significant element of emotional support for women appeared to be rapport building (Spearman’s Rho correlation with SCIB score .432**, p = .002).

**Table 9:** Correlations between positive behaviours and assessments of support

**Midwife behaviours**	**Spearman’s rho**	**Woman’s SCIB score**	**Observer’s global rating of quality**
Emotional support	Correlation	.299*	.505**
	P value	.029	.000
Informational support	Correlation	.248	.364**
	P value	.059	.009
Tangible support	Correlation	.195	.307*
	P value	.111	.024
Support for partner	Correlation	.428**	.376**
	P value	.003	.007
Advocacy	Correlation	-.210	.089
	P value	.094	.285

(** = Significance at p value of <0.01, * = Significance at p value of <0.05).

### Construct validity of the SMILI

The main method to test the construct validity of the instrument is to identify whether women’s views of the support correlate significantly with the data collected using the SMILI. These links have been clearly identified in the previous section, with significant correlations between women’s views and the quantity of positive and negative behaviours recorded.

However, women’s views of the support they received may not only be influenced by the supportive and non-supportive behaviours recorded using the SMILI, but may be also significantly influenced by other elements of the experience. For example, a woman may describe the support she received less positively if she has had a more difficult labour and birth experience in terms of more medical interventions. These other possible influences and their impact on women’s and observers’ views of the support provided were examined.

This analysis found that there were no correlations between women’s views of the support they received and their parity, allocated care pathway, analgesia used, number of medical interventions, type of birth, amount of non-support care and assessment activities, maternity unit and number of years the midwife had been qualified. This may be seen as further evidence to support the construct validity of the SMILI.

## Discussion

This study found that the SMILI was feasible and acceptable for use in the intrapartum setting. The SMILI had been found to have good face and content validity and reliability when tested in a non-clinical setting, this study aimed to identify whether it was reliable, valid and feasible for use in the real world of care of women in labour.

The study was successful in recruiting participants across the four participant sites and achieved the maximum goal for numbers with forty nine complete observations analysed. Overall the great majority of women, birth partners and midwives approached gave consent to participate.

Logistical issues, relating to researcher travel and the smaller numbers of prospective participants meant that recruitment at the two smaller participating units was lower than the larger units.

The study compares favourably in size with the most comparable studies undertaken in North America [11,17-20]. The Miltner study [11] undertook 150 hours of observations of 24 nurses caring for 75 women, the Barnett [20] study undertook 75 hours of observations of 17 nurses caring for 30 women.

The study demonstrated that the direct observational approach was understandable and acceptable to the majority of women, their birth partners and midwives in the clinical setting. The SMILI worked well in the clinical setting with loss of data in only three cases due to user error. The SMILI worked from an inexpensive memory stick on standard personal computer laptops without any special adaptation and so did not represent a costly method of collecting data yet it was successful in obtaining a large amount of comprehensible and usable data relating to intrapartum midwifery care.

The study further tested the SMILI for face, content and construct validity and internal reliability and found the instrument to be both valid and reliable for use in intrapartum care.

The observers rated face and content validity of the SMILI high in the clinical setting. Following some minor amendments to content after the early observations, content validity of the SMILI was found to be high by the observers using the instrument in the setting for which it was designed. The observers felt that the programme enabled them to record the support seen.

The SMILI was found to have good levels of construct validity through the identification of significant correlations between the care recorded and women’s feelings expressed in the postnatal questionnaire (SCIB). Women rated the support they received more highly when they received higher quantities of emotional, informational, tangible and partner support and when the midwife was in the room more. A larger sample of observations would enable further testing of the correlations between both negative and positive behaviours and women’s views. Emotional support behaviours had the strongest links with women’s views, which confirms the findings of earlier studies with women which identify emotional support behaviours as the most important category of support [9,28]. Construct validity of the SMILI was further supported by the findings showing no correlation between the amount of non-support behaviours and non-supportive care including assessment and women’s views of the support received with women’s views in the postnatal questionnaire.

Good levels of internal reliability of the SMILI were found. Different aspects of ‘attitude’ correlated highly with each other for the woman, her birth partner and the midwife. Where a midwife remained in close proximity to the woman, she was also likely to demonstrate a positive demeanour, use a positive vocal tone and display a positive facial expression. Where a midwife displayed a neutral or negative demeanour she was more likely to use a neutral or negative vocal tone and display a negative facial expression.

The veridicality of the methodology was demonstrated through the identified correlation between women’s views of the support received and the observers’ overall assessments of the support they had observed. For the most part, the women and midwives appeared to share their definition of the construct of support. The high level of correlation between the women’s and observers’ assessments further supports the validity of the systematic observational approach to measure the quantity and quality of intrapartum support.

Testing of the inter-rater reliability of the SMILI in the clinical setting was not undertaken. This was due to the judgement that asking women and midwives to consent to have two observers present in the labour room would present more ethical issues in terms of being considerably more invasive of such a private experience.

The SMILI was tested for key features of validity and reliability using a number of approaches and was found to demonstrate good levels of validity and reliability. However, it is recognised that the validity and reliability of any new instrument is an ongoing process, not completed in one study. Further testing of the validity and reliability of the SMILI in other settings will be required to ensure transferability of the instrument to settings outside the specific circumstances of these studies.

The study found that the SMILI and other outcome measures enabled measurement of key aspects of the quantity and quality of midwifery intrapartum support.

The data relating to the quantity of behaviours show considerable variations between midwives and identify some patterns of behaviour for particular midwives. For example, two midwives were found to have shown below the study average of all neutral and negative behaviours and above the study average for all positive support behaviours.

Measuring the quantity of behaviours facilitates the measurement of the quality of the support. The behaviours chosen to be measured using the SMILI were based on the theoretical definitions of high quality intrapartum support and the large body of research undertaken with women that identified the key behaviours they experienced as being supportive and unsupportive. The SMILI demonstrated good sensitivity to differences in behaviour by midwives that resulted in different assessments of support by women and observers. It is therefore possible to begin to build a clearer picture of the details of what may be described as high quality midwifery support.

The widely recognised Institute of Medicine definition of quality in healthcare is care that is person-centred, safe, effective, efficient, equitable and timely p3 [47]. Through the measurement of the amount of time that the midwife is present in the labour room the SMILI is able to contribute to the assessment of the quality of the support through recording the extent to which the support is safe, equitable and timely. Through the measurement of the frequency that negative and positive behaviours are displayed the SMILI is able to assist in the assessment of how person-centred and safe the care is. Through the exploration of correlations between the behaviour data recorded and key clinical outcomes the SMILI can contribute to the assessment of whether the support is safe, effective and efficient.

### Limitations of the research

All of the observations were undertaken in Scotland in NHS hospital settings which may limit the generalisability of findings. The practice of midwifery in the NHS in Scotland may differ in some key respects from other parts of the UK and internationally. While midwives in this study were able to provide one to one support during labour this may not reflect the situation for all midwives at all times providing care in other parts of the NHS in Scotland or elsewhere. However, the aim of this study was to test the validity and reliability of the SMILI in measuring the quantity and quality of midwifery support in labour. The instrument could then be used in a wider range of practice settings to describe routine midwifery support across the UK. While the woman and midwife participants in the study provide a reasonably representative sample of the UK population in terms of risk factors, parity, age, use of pain relief, type of birth, type of midwifery training and years of experience, they do not comprise a representative sample of the UK population in terms of ethnic diversity.

A further potential limitation of the clinical study is the unknown impact of the presence of an observer in the labour room on the quantity and quality of the care provided. Though careful precautions were taken in this study to ensure that the observer was as unobtrusive as possible, the midwives being observed had been made aware of the overall purpose of the research and that the observer would be recording the supportive elements of care. The effect of an observer or researcher on participants cannot be measured. It is possible that the quality of care provided by the observed midwives was on occasion of a higher level than may be expected with no observer present. However, the identification of substantial variations in the behaviours of the midwives and the presence of some negative behaviours suggest that the observers were successful in being unobtrusive and limiting their impact on the care provided.

Finally, accurate consent rate numbers and reasons for non-participation were not collected in the clinical study and would be required in a larger study. Consent was not sought from all women, birth partners and midwives potentially available to be observed on any shift and therefore a true denominator for a consent rate would have been difficult to identify. As midwives who were potential participants were also on occasion seeking the consent of the woman and her birth partner, they operated in some respects as a gatekeeper for entry into the study. The researcher was not present during this information and consent discussion. Any data about rates and reasons for women and birth partners declining participation were therefore ‘filtered’ via the midwives. A data collection procedure for the collection of the number of women, birth partners and midwives approached, number who declined and reasons given for declining would be included in a future study.

## Conclusions

The study described in this paper has made a significant contribution through the development and testing of a robust evidence-based systematic observation instrument. The instrument has been found to be valid, reliable, feasible and usable in intrapartum care. The research has contributed to our knowledge of the intrapartum support provided by midwives currently working in the NHS in Scotland, UK. The study design employed in the research has the potential to provide answers to key gaps in knowledge of intrapartum support and its impact on birth outcomes.

The completion of 104 hours of direct observation of 49 labour episodes represents the largest study of intrapartum support conducted in the United Kingdom.

The SMILI enables an observer in a labour room to record systematically a number of key aspects of the labour episode, including the attitude, demeanour and behaviours of the woman, her birth partner and the midwife. It enables the care observed to be placed in the context of the setting in which it took place. The SMILI produces large amounts of data relating to the presence and absence of the midwife from the room, the frequency of positive supportive behaviours, the frequency of negative behaviours and the frequency of other care activities. These data can be analysed successfully to produce meaningful data to allow comparisons between midwives and between different maternity settings.

The postnatal data collected enable connections to be sought between the quantities of particular behaviours seen and key outcomes, thus enabling meaningful assessments about the quality of the support to be made. Women participants appeared to value emotional support, particularly rapport building, most highly in the care they received.

The Supportive Midwifery in Labour Instrument has the potential to make a substantial contribution in future large scale observational or experimental studies of intrapartum support. The standardised quantitative approach to data collection will enable comparison of results at many levels: between individual practitioners, between different care providers, between institutions and between maternity care systems. The identification of associations between particular support and non-support behaviours and clinical outcomes in such large trials would contribute significantly to the development of labour support theory, the definition of high quality support and the identification of the mechanism of action of support in improving outcomes and promoting normal birth.

The study design facilitates the measurement of the quantity and quality of midwifery support in labour. Through this measurement new knowledge about the nature and impact of support has been and will be generated. This new knowledge can then contribute to the improvement of the quality of support given to women in labour:

‘It is only with careful and systematic inquiry about the nature of midwifery care that the profession can clearly define and explicate a model of excellence that can be upheld as a standard for all women’ p4 [49].

## Abbreviations

NHS Scotland: the National Health Service of Scotland, UK. Health care is provided free at the point of access; SMILI: The Supportive Midwifery in Labour Instrument. The central data collection instrument for the study; SCIB: The Support and Control in Birth questionnaire. A postnatal questionnaire completed by women participants

## Competing interests

The authors declare that they have no competing interests.

## Authors’ contributions

MR-D was the principal investigator for the study and main author of this paper. HC was the principal academic supervisor for Mary Ross-Davie during her doctoral studies. HC provided substantial comments and corrections on the paper. KN was academic supervisor for the doctoral study and contributed significantly to the development of the design of the study. All authors read and approved the final manuscript.

## Pre-publication history

The pre-publication history for this paper can be accessed here:

http://www.biomedcentral.com/1471-2393/13/163/prepub
